# Ammoniagenic Action of Valproate without Signs of Hepatic Dysfunction in Rats: Possible Causes and Supporting Evidence

**DOI:** 10.3390/biom14030370

**Published:** 2024-03-19

**Authors:** Gubidat Alilova, Lyudmila Tikhonova, Carmina Montoliu, Elena Kosenko

**Affiliations:** 1 Laboratory of Cell Engineering, Institute of Theoretical and Experimental Biophysics of Russian Academy of Sciences, 142290 Pushchino, Russia; i@alilova-g.ru (G.A.); ljudasik09@mail.ru (L.T.); 2 Hospital Clinico Research Foundation, INCLIVA Health Research Institute, 46010 Valencia, Spain; 3 Pathology Department, Faculty of Medicine, University of Valencia, 46010 Valencia, Spain

**Keywords:** valproate, ammoniagenesis in the rat brain, encephalopathy without liver failure

## Abstract

(1) Background: Valproic acid (VPA) is one of the frequently prescribed antiepileptic drugs and is generally considered well tolerated. However, VPA neurologic adverse effects in the absence of liver failure are fairly common, suggesting that in the mechanism for the development of VPA-induced encephalopathy, much more is involved than merely the exposure to hyperammonemia (HA) caused by liver insufficiency to perform detoxification. Taking into account the importance of the relationship between an impaired brain energy metabolism and elevated ammonia production, and based on the ability of VPA to interfere with neuronal oxidative pathways, the current study intended to investigate a potential regional ammoniagenic effect of VPA on rats’ brains by determining activities of the enzymes responsible for ammonia production and neutralization. (2) Methods: Rats received a single intraperitoneal injection of VPA (50, 100, 250, 500 mg/kg). Plasma, the neocortex, the cerebellum, and the hippocampus were collected at 30 min after injection. The levels of ammonia, urea, aspartate aminotransferase (AST), and alanine aminotransferase (ALT) were measured in blood plasma. The activities of glutaminase and glutamate dehydrogenase (GDH) in mitochondria and the activities of AMP deaminase (AMPD), adenosine deaminase (ADA), and glutamine synthetase (GS) in cytosolic fractions isolated from rat brain regions were measured. Ammonia, ALT, and AST values were determined in the mitochondrial and cytosolic fractions. (3) Results: Multi-dose VPA treatment did not significantly affect the plasma levels of ammonia and urea or the ALT and AST liver enzymes. Significant dose-independent increases in the accumulation of ammonia were found only in the cytosol from the cerebellum and there was a strong correlation between the ammonia level and the ADA activity in this brain structure. A significant decrease in the AMPD and AST activities was observed, while the ALT activity was unaffected. Only the highest VPA dose (500 mg/kg) was associated with significantly less activity of GS compared to the control in all studied brain structures. In the mitochondria of all studied brain structures, VPA caused a dose-independent increases in ammonia levels, a high concentration of which was strongly and positively correlated with the increased GDH and ALT activity, while glutaminase activity remained unchanged, and AST activity significantly decreased compared to the control in all studied brain structures. (4) Conclusions: This study highlights the rat brain region-specific ammoniagenic effects of VPA, which may manifest themselves in the absence of hyperammonemia. Further research should analyze how the responsiveness of the different brain regions may vary in VPA-treated animals that exhibit compromised energy metabolism, leading to increased ammoniagenesis.

## 1. Introduction

Valproic acid (VPA), which has a broad spectrum of efficacy against all types of seizures, is widely used as a first-line anticonvulsant for the treatment of epileptic syndromes and other psychiatric disorders in children and adults, and is generally considered well tolerated [1].

However, many adverse effects related to the use of VPA in clinical settings are reported in the literature [2,3]. One of the VPA-induced life-threatening side effects is acute hyperammonemia (HA) (an excess amount of ammonia in the blood) allowing the fast access of blood ammonia to the brain, which leads to the development of encephalopathy, accompanied by a loss of consciousness, seizures, and comas, which often have a fatal outcome [4,5,6]. Also, it has been reported that VPA-induced comas occur regardless of normal serum VPA levels [7], suggesting that neurotoxic metabolites of VPA [8] are responsible for at least part of the spectrum of CNS depression [2]. Despite numerous studies undertaken to elucidate the exact mechanism of the observed effects, no clear evidence has been established.

It is also worth noting that ammonia, which is formed constantly in tissues of the body, is an intracellular metabolite that performs many vital functions [9] but exhibits neurotoxic properties very rapidly when its levels in the blood increase. Therefore, it is not surprising that although ammonia formation is taking place continuously in the cells, the steady-state ammonia concentration in human tissues under physiological conditions varies in a narrow range, and the blood ammonia content is maintained at a very low level not exceeding 50 μmol/L [10]. This is achieved due to the urea cycle enzymes, which are located exclusively in the liver, which detoxify ammonia, and due to another liver enzyme, glutamine synthetase (GS), which converts ammonia that does not enter the urea cycle into relatively harmless glutamine. In other organs, for example in the brain that does not contain the urea cycle, GS is the only enzyme that can detoxify ammonia. Thus, HA can occur as a nonspecific response to any liver injury or due to a portosystemic shunt enabling the circulating of blood with a significant amount of ammonia from the gastro-intestinal tract to bypass the liver. As a result, ammonia circulating in the blood can reach the systemic circulation, thereby obtaining easy access to the brain [11], resulting in ammonia-induced encephalopathy [12]. The validity of the concept is not in doubt, but the established relationship of “liver damage → HA → encephalopathy” in the analysis of the known mechanisms underlying VPA-induced HA raises a number of questions.

According to available data, VPA and its metabolites can cause HA through several mechanisms. The most important route appears to be the inhibition of carbamoyl phosphate synthetase-1, the first enzyme of urea synthesis, which resides in liver mitochondria [13] and other processes in the mitochondrial membrane [14] and in the mitochondrial matrix [15], which directly or indirectly reduces the availability of ATP required for urea production [16,17]. However, these data cannot explain why under the conditions when VPA inhibits the urea cycle in the liver, the urea concentration found in the blood of some patients remains within normal limits [18,19,20,21,22,23].

The questions also arise as to why does VPA-induced encephalopathy occur more often in patients without a history of the liver disease but with normal liver function tests [21,24,25,26] and what is the role of the liver in the absence of hepatic failure in case of hyperammonemia-induced encephalopathy? Lastly, if VPA causes encephalopathy in some patients who have normal ammonia levels in the blood (non-hyperammonemic valproate-induced encephalopathy), the symptoms of which rapidly improve after stopping VPA [27,28,29,30], what role does the liver play in non-hyperammonemic valproate-induced encephalopathy? The more data researchers obtained, the more it became apparent that increased blood ammonia levels if the liver is unable to filter the blood do not actually cover the full spectrum of causes of adverse reactions to VPA in terms of encephalopathy. To highlight the diversity of possible causes, this study aimed at exploring alternative causes of VPA-induced ammonia formation leading to neurotoxicity and encephalopathy that are unrelated to liver pathology.

Our previous study showed that in rats with a portacaval shunt, when the concentration of ammonia is doubled in the blood, its concentration in different brain regions was five to six times higher than that in plasma [31]. The detected gradient in the concentration of ammonia (low in plasma and much higher in the brain) suggests that ammonium ions are unlikely to be consumed by the brain tissue from plasma but are formed in the brain as a result of the activation of endogenous ammonia-forming reactions. Consequently, it is thought that the endogenous accumulation of ammonia in the brain can promote seizures and comas regardless of the ammonia concentration in the blood. This conclusion is shared by the findings of Hoyer [32], who showed that under a reduction in the cerebral metabolic rate of glucose in patients with Alzheimer’s disease, a significant amount of the ammonia can be produced in the brain, as well as be released into the blood [33]. Thus, disturbances in the energy balance may lead to high concentrations of ammonia in the brain [32,34]. Based on the partly assessed role of a VPA-associated compromise in the neuronal oxidative metabolism [15,35], it is assumed that VPA may be a trigger for ammonium production in the brain.

To date, no research has been conducted to perform a systematic review to find answers to the research question of whether VPA has an effect on ammoniagenesis in the animal brain under experimental conditions without a history of liver disease. Therefore, the objective of our study was to elucidate whether VPA promotes the accumulation of endogenous ammonia beyond normal physiological thresholds in the rat brain.

Given the above as well as the fact that different areas of the brain may differ in their ammonia production and clearance abilities [36], a key objective of our research process was to determine whether VPA exhibits its effect on a homeostatic system of ammonia and examine various enzymatic systems involved in ammonia formation and neutralization in the mitochondria and cytosol of various rat brain regions.

## 2. Materials and Methods

### 2.1. Materials

The chemical reagents used were purchased/obtained from Sigma Chemical Co. (St. Louis, MO, USA) and were as follows: VPA, Tris, HEPES, NAD^+^, NADH, ATP, AMP, GSH, sucrose, mannitol, glutamate, glutamine, adenosine, GDH, EGTA, EDTA, phenylmethylsulfonyl fluoride, dithiothreitol, leupeptin, pepstatin, aprotinin A, and rotenone. Other reagents used were of the highest purity attainable.

### 2.2. Experimental Section

#### 2.2.1. Experimental Design

This study was conducted in accordance with the ethical principles underlying the Declaration of Helsinki for animals as an ethical ‘best practice’ in clinical veterinary research and the Regulations of the European Science Association (revised in the European Directive 86/609/EEC and formulated in the Order of the Ministry of Health of the Russian Federation of 19.06.2003.267 “Guidelines on good laboratory practice”).

#### 2.2.2. Animals

Male Wistar rats weighing 200–220 g were used. The animals were fed a standard chow diet supplemented by ad libitum access to food and water. In the experiment, rats were housed at an ambient room temperature of 22 °C.

As VPA is rapidly cleared from the rats’ plasma and transferred into the brain within a few minutes after its administration [37], and its neurological adverse effects and severe HA may develop suddenly [38], even after the application of both defined daily doses or drug overdoses [39,40] the animals (VPA group) were intraperitoneally injected with different single doses of VPA 50, 100, 250, 500 mg/kg.

The doses of VPA were selected on the basis of studies conducted in animal models and were close to concentrations to be used in the treatment of human status epilepticus [41].

As very short-term changes in the rats were observed in neurological and behavioral responses that may persist during the first several minutes of testing and when the variability in the dose was high [42], the animals were decapitated 30 min after VPA injection.

VPA was dissolved in a sterile solution of 0.9% NaCl at a concentration of 400 mg/mL, with pH = 7.5.

Control animals were injected with saline in equal volumes via the same route.

All experiments were carried out at 10:00 a.m. to avoid bias introduced by circadian variations in enzyme activities and metabolite levels.

#### 2.2.3. Preparative and Analytical Methods

Blood was drawn from the retro-orbital plexus into citrate-treated tubes. Plasma obtained by the standard method was deproteinized with a cold mixture (–20 °C) of 6% HClO_4_ and 40% ethanol and neutralized with KOH to pH 6. KClO_4_ crystals were precipitated by centrifugation, and the resulting supernatant was immediately used to determine the plasma ammonium and other metabolites and enzymes.

The microfluorometric method was applied to the estimation of ammonia content in blood plasma using a procedure described earlier [31].

The urea concentration was measured after preliminary hydrolysis of the urea with the aid of urease-producing ammonia, the concentration of which was determined in the glutamate dehydrogenase (GDH) reaction.

The 1 mL reaction mixture contained 50 mM KH_2_PO_4_ (pH 7.6), 7 mM α-ketoglutarate, 0.16 mM NADH, 4 U/mL GDH, and 2.5 U/mL urease. The reaction was started by adding 25 μL of plasma. The urea concentration was expressed as mmol/L [43].

Aspartate aminotransferase (AST, EC 2.6.1.1) and alanine aminotransferase (ALT, EC 2.6.1.2) activities were measured by spectrophotometry using the commercial MAK0551KT assay kit (Sigma-Aldrich, Saint Louis, MO, USA).

Brains were removed (20 s after decapitation), and the neocortex, hippocampus, and cerebellum were separated and placed in ice-cold buffer containing 10 mM Tris, at pH 7.4, with 0.25 M sucrose, and 0.5 mM EGTA (TSE buffer). Brain tissues were chopped with scissors, following frequent washing in cold TSE buffer to remove traces of blood, and then homogenized with nine volumes of the same buffer. The homogenate was centrifuged twice at 2000× *g* for 3 min, and the resulting supernatant was centrifuged at 12,000× *g* for 8 min to obtain a pellet of mitochondria. After washing, the mitochondrial sediment was suspended in the TSE buffer without EDTA.

The post-mitochondrial supernatant was centrifuged at 100,000× *g* at 4 °C for 30 min and the resulting supernatant was used as a cytoplasmic fraction.

To determine the activity of intramitochondrial enzymes, mitochondria were disrupted by osmotic shock in 5 mM phosphate buffer, at pH 7.4, containing 0.1 mM EGTA, 0.5 mM phenylmethylsulfonyl fluoride, 1 mM dithiothreitol, 10 μM leupeptin, 1.5 μM pepstatin, and 0.15 μM aprotinin A at 4 °C for 15 min followed by three freeze–thaw cycles.

Glutaminase activity (EC 3.5.1.2) was determined spectrophotometrically following the formation of glutamate from glutamine [44].

The mitochondrial protein (0.1 mg) was incubated in a medium containing 150 mM potassium phosphate and 50 mM Tris-HCl, at pH 8.6, with 0.2 mM EDTA, 10 mM glutamine, and 1 mM rotenone at 37 °C. After 10 min of incubation, the reaction was stopped by the addition of 0.1 mL of ethanol. The concentration of glutamate, formed in the reaction, was measured in the GDH reaction by NAD^+^ reduction at 340 nm.

Glutamate dehydrogenase (GDH, EC 1.4.1.3) activity was assayed spectrophotometrically at 340 nm and 37 °C following NADH oxidation. The enzymatic reaction was initiated by the addition of 40 μg of protein [45].

Glutamine synthetase (GS, EC 6.3.1.2) activity was measured using the method of Meister [46], using 20 μg of cytoplasmic protein in a total volume of 1 mL.

The analysis of AST (EC 2.6.1.1) and ALT (EC 2.6.1.2) was performed spectrophotometrically using the commercial MAK0551KT assay kit (Sigma-Aldrich, USA) and 20–80 μg of cytoplasmic or mitochondrial protein.

The activity levels of AMP deaminase (AMPD, EC 3.5.4.6) and adenosine deaminase (ADA, EC 3.5.4.4) in the cytoplasmic fractions of the brain were determined spectrophotometrically at 340 nm and 37 °C following the formation of ammonia and the oxidation of NADH in the coupled GDH reaction as described previously [47]. The AMPD reaction was initiated by the addition of 5 mM AMP; the ADA reaction was started by adding 1 mM adenosine.

The protein in subcellular fractions was determined according to Lowry et al. [48].

### 2.3. Statistical Analysis

The statistical processing of the results was performed using the program Prizm 8.0.1. for Windows (GraphPad Software, San Diego, CA, USA). The normal distribution of variables was confirmed using the Kolmogorov–Smirnov test. Pairwise comparisons were carried out using Student’s *t*-test, and multiple comparisons were performed using the ANOVA and Bonferroni corrections. The results were expressed as mean ± standard error of the mean (SEM). *p* < 0.05 was considered significant. To assess whether cytosolic and mitochondrial ammonia levels increased with an increase in registered activities of ammonia-generating enzymes, a correlation analysis was performed using linear regression and Pearson’s correlation coefficient. *p* values < 0.05 were considered to be statistically significant.

## 3. Results

### 3.1. Ammonia, Urea Concentration, and Alanine Aminotransferase and Aspartate Aminotransferase Activities in the Plasma of Rats

To determine whether VPA could affect the liver’s capacity to clear ammonia and alter the integrity of the liver cells, it was important to have an understanding of whether the concentrations of ammonia and urea as well as the activities of alanine aminotransferase (ALT) and aspartate aminotransferase (AST) (classical “liver tests”) changed in the blood plasma of the rats injected with different single doses of VPA (Table 1).

As shown in Table 1, acute treatment with VPA did not significantly affect the activities of the liver enzymes such as ALT and AST as well as the plasma concentrations of ammonia and urea in the animals. All values in the rat plasma were closely proportional to those obtained from the control group, but were not correlated with the animals’ behaviors aberrant as per the form or frequency.

Our findings suggest that acute VPA administration, even in a maximum dose (500 mg/kg), had no effect on the capacity of the liver to detoxify ammonia and caused no alteration of the integrity of cells in the liver, and that the neurological effects (depending on the dose and thus high variable) observed in the drug-treated animals (such as increases in locomotor activity and anxiety giving way to lower exploratory activity, slackness, drowsiness, tremors, and myoclonic seizures) may develop regardless of HA and liver pathology.

### 3.2. The Effect of Different Doses of VPA on the Concentration of Ammonia in the Mitochondria and Cytosol in Different Rat Brain Regions

In vivo studies indicated that VPA causes a dramatic increase in glutamine in the brain of patients with epilepsy [49] and in most of the models of generalized seizures in rodents [50]. Glutamine is a byproduct of ammonia metabolism and its accumulation in the cytosol and mitochondria of the brain cells can affect brain ammonia homeostasis [51]. It was therefore possible that when penetrating the brain, VPA directly or indirectly disrupts the balance between the rate of ammonia production and the rate at which it is disposed of, and thus contributes to the accumulation of neurotoxins in the brain.

**Figure 1:**
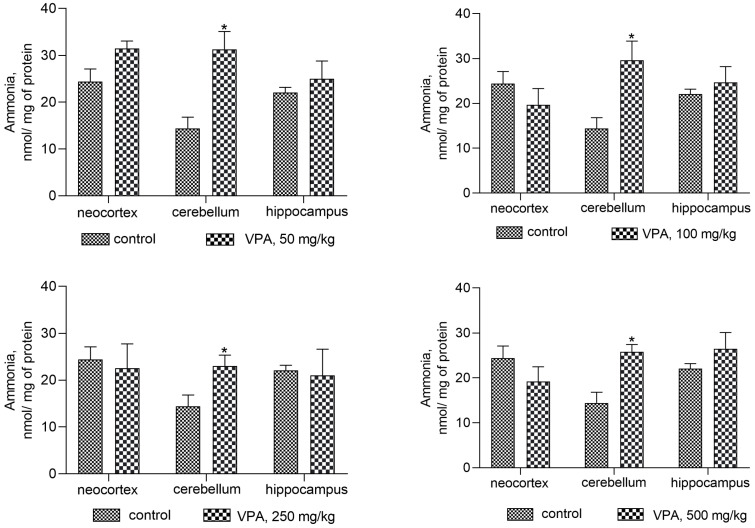
The concentrations of ammonia in the cytosol fraction of the different rat brain regions. The animals in the VPA group received i.p. injection of different single doses of VPA in 0.9% NaCl. Animals in the control group were given an equal volume of saline by the same route. The animals from these two groups were decapitated 30 min after VPA or saline injection. The results are the mean ± SEM of 8 rats per group. Values that are significantly different from those of the control group are marked with an asterisk: * *p* < 0.05 (with the Bonferroni correction for multiple comparisons).

**Figure 2:**
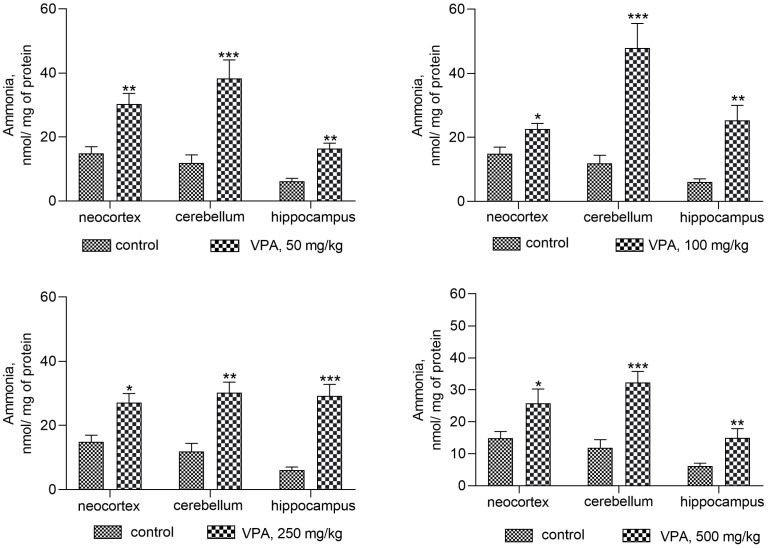
The concentration of ammonia in the mitochondria of different rat brain regions. The animals in the VPA group received i.p. injection of different single doses of VPA in 0.9% NaCl. Animals in the control group were given an equal volume of saline by the same route. The animals from these two groups were decapitated 30 min after VPA or saline injection. The data are expressed as the mean ± SEM of 8 rats per group. Values that are significantly different from those of the control group are marked with an asterisk: * *p* < 0.05, ** *p* < 0.01, *** *p* < 0.001 (with the Bonferroni correction for multiple comparisons).

To confirm this assumption and to identify an additional mechanism for VPA to participate in the increased production of cerebral ammonia, first this study examined how a single injection of various doses of VPA affects the ammonia levels in the cytosolic fractions (Figure 1) and mitochondria (Figure 2) isolated from the various regions of the animals’ brain.

Our findings demonstrate that the effects of the drug on ammonia levels in brain regions differ markedly (Figure 1). Figure 1 shows a significant increase of ammonia levels in the cytosolic fraction after VPA injection, when compared to the control (14.4 ± 2.4 nmol/mg protein), only in the cerebellum. The measured levels of ammonia in this brain region did not change with the dose and were as follows: 31.3 ± 3.8, 29.6 ± 4.3, 23.03 ± 2.3, and 25.83 ± 1.6 nmol/mg protein for doses of 50, 100, 250, and 500 mg/kg, respectively. No significant VPA effect on ammonia levels was seen in the other brain areas (Figure 1).

In the mitochondria (Figure 2), on the contrary, valproate increased the accumulation of ammonia in all investigated brain structures, but again its effect was not dose-dependent.

**Figure 3:**
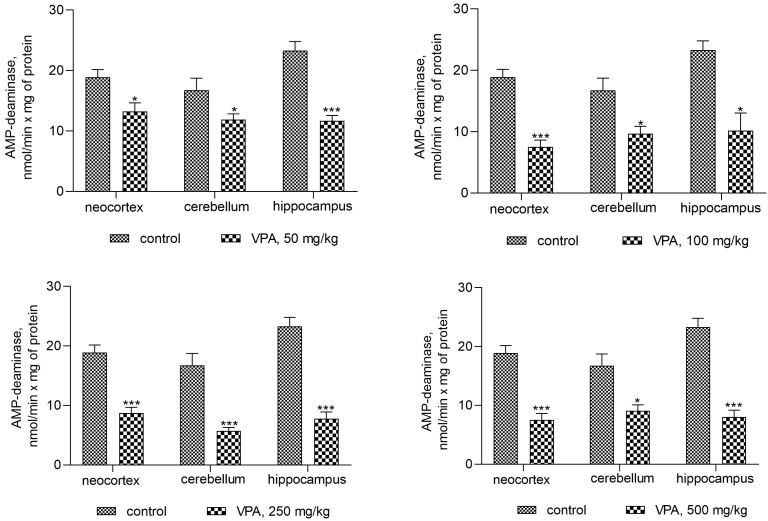
The effect of a single intraperitoneal administration of VPA (at four doses of 50 mg/kg, 100 mg/kg, 250 mg/kg, and 500 mg/kg) on AMP-deaminase activity in the cytosolic fraction isolated from different brain regions. The animals in the two groups were decapitated 30 min after VPA or saline injection. The results are the mean ± SEM of 8 rats per group. Values that are significantly different from those of the control group are marked with an asterisk: * *p* < 0.05, *** *p* < 0.001 (with the Bonferroni correction for multiple comparisons).

The values of ammonia in the mitochondria isolated from the neocortex, cerebellum, and hippocampus of the control animals according to Figure 3 were 14.87 ± 2.08, 11.92 ± 2.5, and 6.1 ± 0.97 nmol/mg protein, respectively.

The minimum dose of VPA at 50 mg/kg caused a 2–3-fold increase in ammonia levels, and there was no additional accumulation of ammonia in all types of mitochondria at the highest dose of VPA at 500 mg/kg (Figure 2).

The results obtained showed that acute exposure to VPA disrupts ammonia homeostasis in the brain and that this specific adverse effect of VPA is associated with increases in ammonia levels in cytosolic (only in the cerebellum) and mitochondrial fractions isolated from the rat neocortex, cerebellum, and hippocampus, as the majority of other side-effects of VPA are dose-independent [52,53,54].

### 3.3. The Effect of Valproate on AMP-Deaminase and Adenosine Deaminase Activities in the Cytosol of Different Rat Brain Structures

To further elucidate whether changes in the levels of ammonia happened at the same time as changes in enzymes involved in its endogenous brain formation and inactivation, the effects of increasing doses of VPA on the specific activity of AMP deaminase (AMPD) and adenosine deaminase, the enzymes of the purine nucleotide cycle, as well as ALT, AST, and glutamine synthetase were examined in cytosolic fractions of the rat brain neocortex, cerebellum, and hippocampus.

The activities of AMPD are presented in Figure 3.

Statistical analysis with Bonferroni’s correction revealed a significant decrease in AMPD activity in all investigated brain regions of VPA-treated animals as compared to the control group.

Minimal decreases in the enzyme activity of 30% (* *p* < 0.05), 29% (* *p* < 0.05), and 50% (*** *p* < 0.001) were present in the neocortex, cerebellum, and hippocampus, respectively, for VPA doses of 50 mg/kg.

Interestingly, it was found that a double dose (100 mg/kg) of VPA decreased the AMPD activity proportionally to one dose only in the neocortex (60%, *** *p* < 0.001), while in other brain structures, the magnitude of the activity of the enzymes remained unchanged upon application of all VPA doses (on average 42–67% as compared to the control) (Figure 3). Our evidence indicates that the minimum inhibitory dose of VPA may be lower than 50 mg/kg.

**Figure 4:**
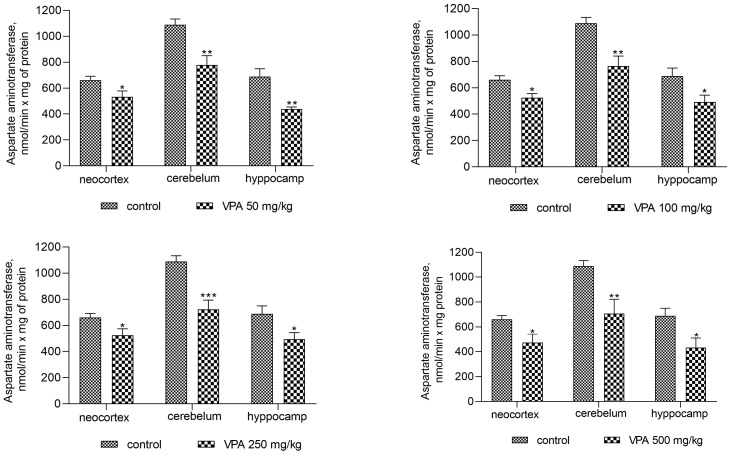
The effect of a single intraperitoneal administration of VPA (at four doses of 50, 100, 250, and 500 mg/kg) on AST activity in the cytosolic fraction isolated from different brain regions. The animals in the two groups were decapitated 30 min after VPA or saline injection. The data are expressed as the mean ± SEM of 8 rats per group. Values that are significantly different from those of the control group are marked with an asterisk: * *p* < 0.05, ** *p* < 0.01, *** *p* < 0.001 (with the Bonferroni correction for multiple comparisons).

The activity of AST was also reduced in all investigated brain regions after sodium valproate treatment at all dose levels (Figure 4). The enzyme activity decreased significantly in the cytosol of the neocortex, cerebellum, and hippocampus by 19%, 28.5%, and 31.5%, respectively, when the dose of sodium valproate was 50 mg/kg. The magnitude of the inhibitory effect of VPA was not dose-dependent and was 28.6%, 35%, and 32% for the neocortex, cerebellum, and hippocampus, respectively, at the maximum used dose of VPA of 500 mg/kg (Figure 4).

**Figure 5:**
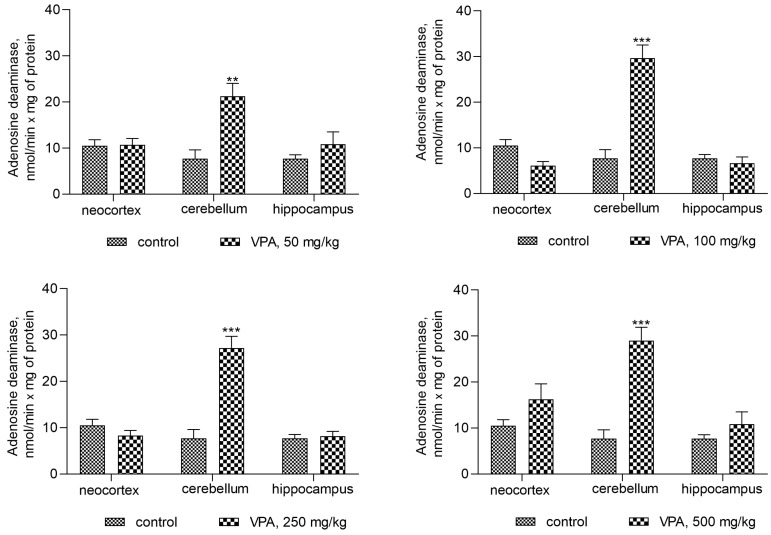
The effect of a single intraperitoneal administration of VPA (at four doses of 50, 100, 250, and 500 mg/kg) on the ADA activity in the cytosolic fraction isolated from different brain regions. The animals from the two groups were decapitated 30 min after VPA or saline injection. The results are the mean ± SEM of 8 rats per group. Values that are significantly different from those of the control group are marked with an asterisk: ** *p* < 0.01, *** *p* < 0.001 (with the Bonferroni correction for multiple comparisons).

Under the experimental conditions used, the activity of the cytosolic ALT in all investigated brain regions was unaffected by VPA as compared to the control (not shown), whereas the activity of adenosine deaminase (ADA), which is directly involved in ammonia synthesis, was significantly increased as compared to the control (7.7 ± 1.9 nmol/min × mg protein) only in the cerebellum of the experimental animals (Figure 5) and amounted to 21.24 ± 2.8 (176%, *p* < 0.01), 29.65 ± 2.9 (285%, *p* < 0.001), 27.2 ± 2.5 (253%, *p* < 0.001), and 29.03 ± 2.9 (277%, *p* < 0.001) nmol/min × mg for doses of 50 mg/kg, 100 mg/kg, 250 mg/kg, and 500 mg/kg, respectively.

Assuming that the ammonia concentrations increased exclusively in the cerebellar cytosol of animals at all administered VPA doses (Figure 5), a correlation analysis between the ADA activity and ammonia levels was performed to determine whether ADA upregulation in this brain region is critical for the accumulation of ammonia.

**Figure 6:**
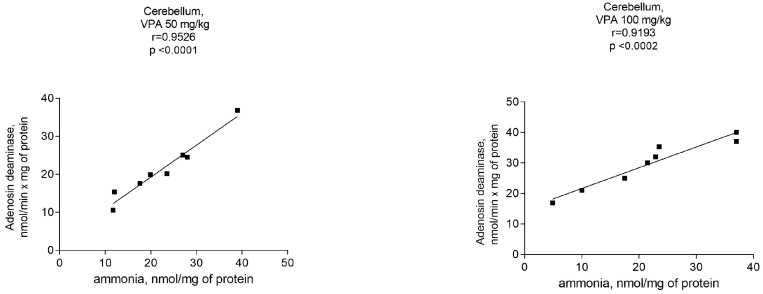

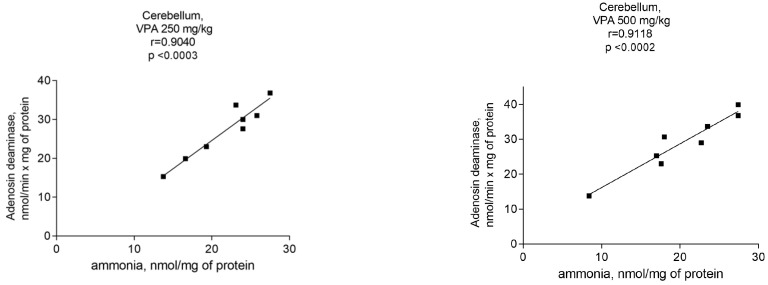
The correlations between ADA activity and ammonia levels in cytosolic fractions of the rat cerebellum after treatment with VPA at different dose levels (*n* = 8). The animals were decapitated 30 min after VPA injection. Specific enzyme activity (nmol/min × mg protein) was positively correlated with ammonia levels (nmol/mg protein) (*r* values represent Pearson correlation coefficients; *r* = 0.904–0.952).

As can be seen from Figure 6, there were positive correlations between the ADA activity and ammonia levels. The Pearson correlation coefficient *r* values, were *r* = 0.9526 (*p* < 0.0001), *r* = 0.9193 (*p* < 0.0002), *r* = 0.9040 (*p* < 0.0003), and *r* = 0.9118 (*p* < 0.0002) at VPA doses of 50 mg/kg, 100 mg/kg, 250 mg/kg, and 500 mg/kg, respectively.

When taken together, these findings suggest that VPA causes region-specific changes in the activities of enzymes involved in ammonia synthesis and metabolism and that the VPA-induced upregulation of ADA in the cytosol of the cerebellum in vivo directly affects the ammonia production rate and contributes to the accumulation of the neurotoxin in this brain region. This is consistent with the fact that the cerebellum, unlike other brain regions, is more closely intertwined under non-physiological conditions with processes related to disturbances of the ammonia homeostasis [36,55,56].

### 3.4. The Effect of VPA on Glutaminase, Glutamate Dehydrogenase, Alanine Aminotransferase, and Aspartate Aminotransferase Activities in the Mitochondria of Different Regions of the Rat Brain

To further elucidate whether the VPA-induced increase in ammonia levels in the mitochondria isolated from the all studied brain regions (Figure 2) occurs in parallel with changes in enzymes involved in ammonia formation in mitochondria, the activities of glutaminase, GDH, ALT and AST have been measured.

**Figure 7:**
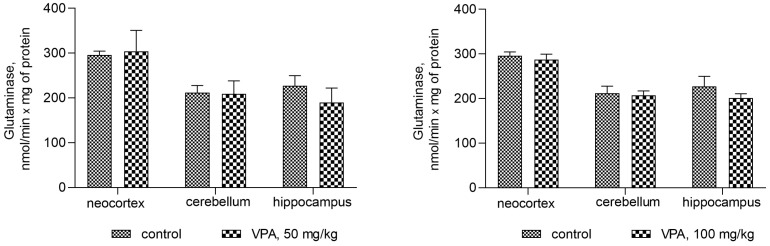

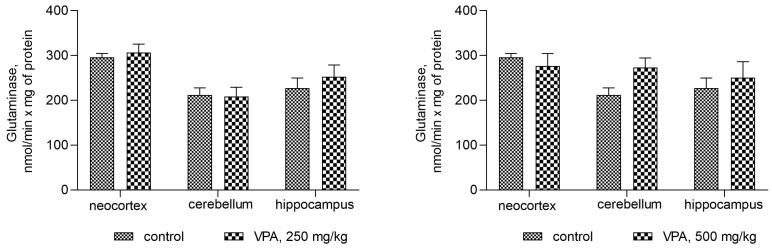
The effect of a single intraperitoneal administration of VPA (at four doses of 50 mg/kg, 100 mg/kg, 250 mg/kg, and 500 mg/kg) on the activity of glutaminase in mitochondria isolated from different brain regions. The animals from the two groups were decapitated 30 min after VPA or saline injection. The results are the mean ± SEM of 8 rats per group.

Figure 7 shows that the administration of the drug at different dose levels had no effect on the activity of glutaminase in the mitochondria from all studied brain regions. Another process related to ammonia production can be accelerated by the concerted reactions of GDH and transaminases, the enzymes involved in the transamination of amino acid with α-ketoglutarate to form glutamate, which is then deaminated and thereby impacts the fate of ammonia in brain mitochondria. The assumption that this pathway is responsible for the increased production of ammonia in the mitochondria of VPA-treatment animals was checked.

**Figure 8:**
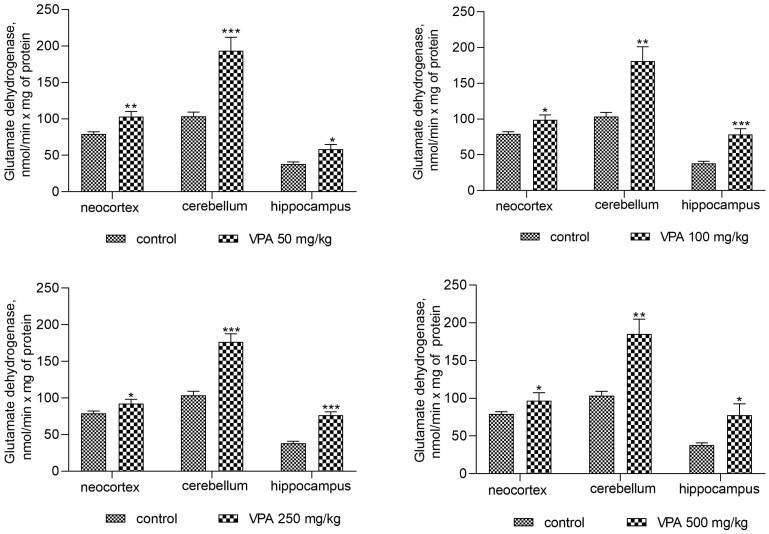
GDH in mitochondria isolated from different brain regions of the animals that received VPA at doses of 50, 100, 250, and 500 mg/kg. The animals from the two groups were decapitated 30 min after VPA or saline injection. The results are the mean ± SEM of 8 rats per group. Values that are significantly different from those of the control group are marked with an asterisk: * *p* < 0.05, ** *p* < 0.01, *** *p* < 0.001 (with the Bonferroni correction for multiple comparisons).

Indeed, the results of our study have demonstrated that such a link really does exist. A VPA dose-independent increase in GDH activity was seen in mitochondria from all brain regions under study (Figure 8). Different doses of VPA at 50 mg/kg, 100 mg/kg, 250 mg/kg, and 500 mg/kg minimally but significantly increased the activity of GDH in the neocortical mitochondria by 33%, 25%, 16.5%, and 23%, respectively, as compared to the control (Figure 8).

**Figure 9:**
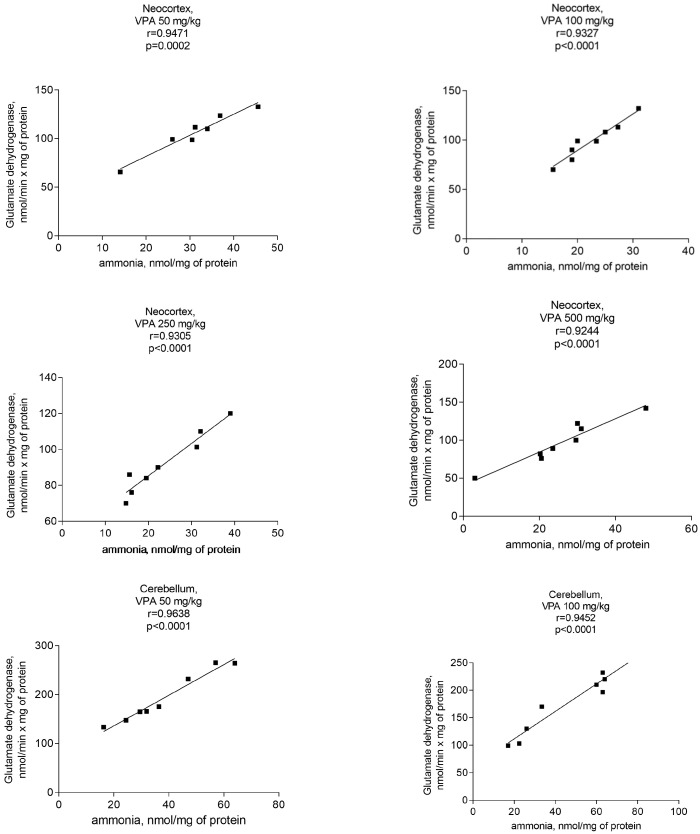

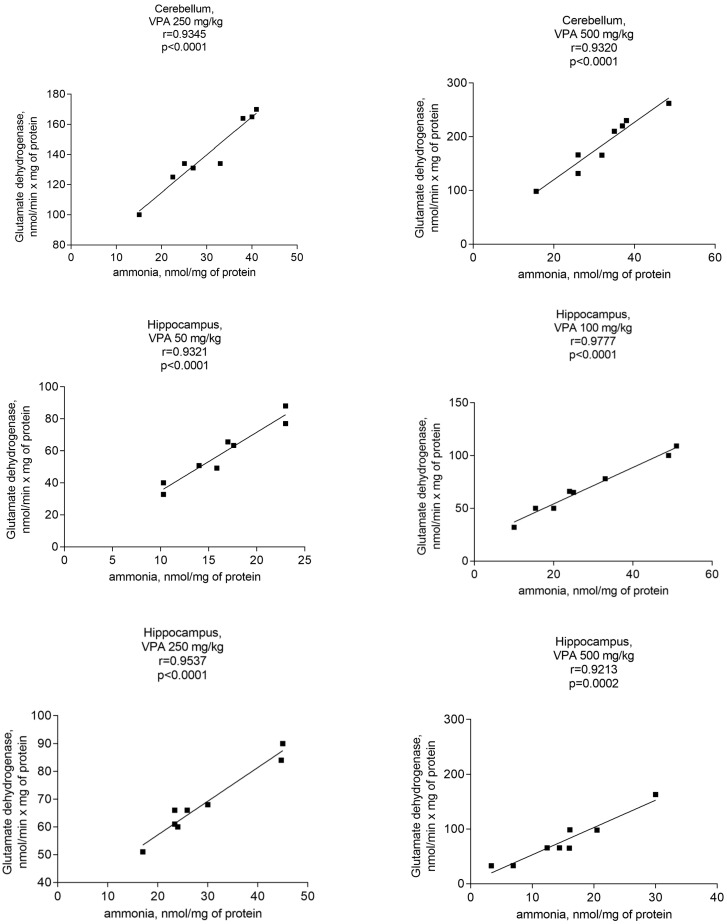
A positive correlation between mitochondrial GDH and ammonia in different brain areas of VPA-administered rats (50 mg/kg, 100 mg/kg, 250 mg/kg, and 500 mg/kg) (*r* = 0.9040–0.97770).

A more pronounced VPA-induced and dose-independent increase in GDH activity was seen in the cerebellar mitochondria. As shown in Figure 9, in these organelles of VPA-exposed rats, we found increases in GDH activity of 87%, 75%, 71%, and 79% at drug doses of 50 mg/kg, 100 mg/kg, 250 mg/kg, and 500 mg/kg, respectively, when compared to the control values (*p* < 0.01–0.001).

The greatest rise (but with no clear linear relationship to the drug dosage) in GDH activity by 54%, 80%, 105.7%, and 105% compared to the control was seen in the hippocampus of the rats injected with 50 mg/kg, 100 mg/kg, 250 mg/kg, and 500 mg/kg VPA, respectively (Figure 8).

Analyses performed to establish a relationship between GDH activity and ammonia concentrations in the mitochondria of VPA-treated rats clearly revealed a correlation between the ammonia accumulation and the activity of the said enzyme.

A significant positive correlation was found between the GDH activity and ammonia levels in the mitochondria of the neocortex, cerebellum, and hippocampus of the animals exposed to various doses of VPA (Figure 9).

The *r* values for all studied brain regions of the animals that received different doses of the drug varied from 0.9040 to 0.9777 (Figure 9) and *p* = 0.0001–0.0002. These findings suggest that VPA may disrupt the ammonium homeostasis by increasing the activity of GDH, and that this enzyme may be the main enzyme responsible for ammonia production in the brain mitochondria of VPA-treated rats.

**Figure 10:**
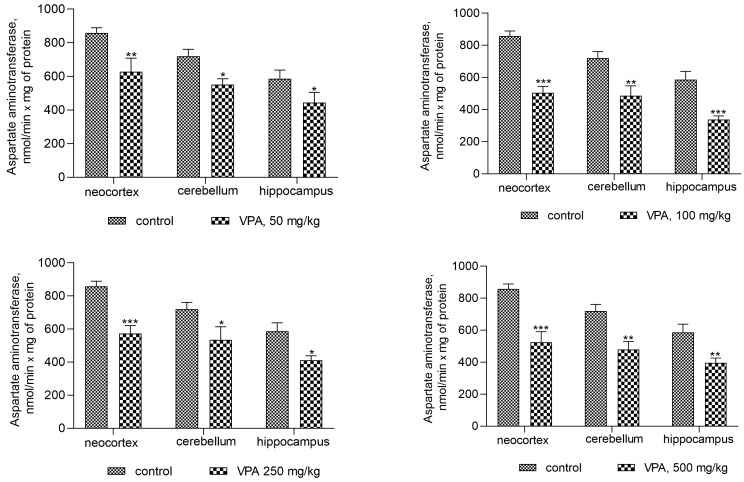
AST in mitochondria isolated from different brain regions of VPA-administered animals (50 mg/kg, 100 mg/kg, 250 mg/kg, and 500 mg/kg). The animals from the two groups were decapitated 30 min after VPA or saline injection. The results are the mean ± SEM of 8 rats per group. Values that are significantly different from those of the control group are marked with an asterisk: * *p* < 0.05, ** *p* < 0.01, *** *p* < 0.001 (with the Bonferroni correction for multiple comparisons).

However, the present study demonstrates that the AST activity was significantly decreased in mitochondria (as in the cytosol, Figure 4) of all studied brain regions after VPA treatment (Figure 10) as compared to control. This corresponds closely to the dynamics of the decrease in the concentration of aspartate in the studied areas of the brain [57]. These data indicate, on the one hand, the direct effect of VPA on AST involved in the synthesis of aspartate [50,57], and, on the other hand, the absence of the expected combined effect of AST and GDH activities followed by the formation of ammonia.

**Figure 11:**
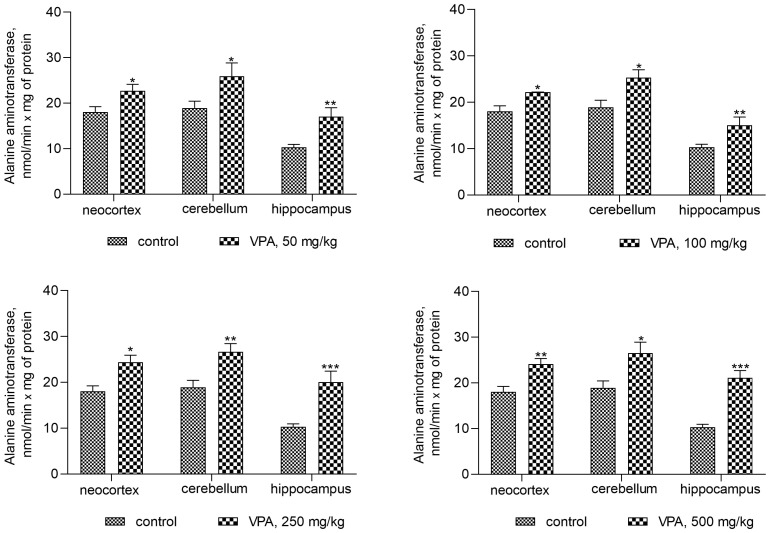
ALT in mitochondria isolated from different brain regions of VPA-administered animals (50 mg/kg, 100 mg/kg, 250 mg/kg, and 500 mg/kg). The animals from the two groups were decapitated 30 min after VPA or saline injection. The results are the mean ± SEM of 8 rats per group. Values that are significantly different from those of the control group are marked with an asterisk: * *p* < 0.05, ** *p* < 0.01, *** *p* < 0.001 (with the Bonferroni correction for multiple comparisons).

On the contrary, the ALT activity in mitochondria isolated from all brain regions of interest increased significantly (Figure 11) when compared to the control. This pattern of change indicates that VPA produced a disproportionate increase in the enzyme activity.

Thus, a minimal and almost equal increase in ALT activity of 22–33% when compared to the control was observed in the neocortical mitochondria (Figure 11) of the rats exposed to different doses of VPA.

In the cerebellar mitochondria, a similar dose-independent increase in the enzyme activity amounted to 37%, 33.6%, 41%, and 40% at doses of 50 mg/kg, 100 mg/kg, 250 mg/kg, and 500 mg/kg VPA, respectively, as compared to the control (Figure 11).

The increase in ALT activity in hippocampal mitochondria was VPA dose-dependent but dose non-proportional and was 65.5%, 73%, 95%, and 105% higher at doses of 50 mg/kg, 100 mg/kg, 250 mg/kg, and 500 mg/kg VPA, respectively, than the control values.

**Figure 12:**
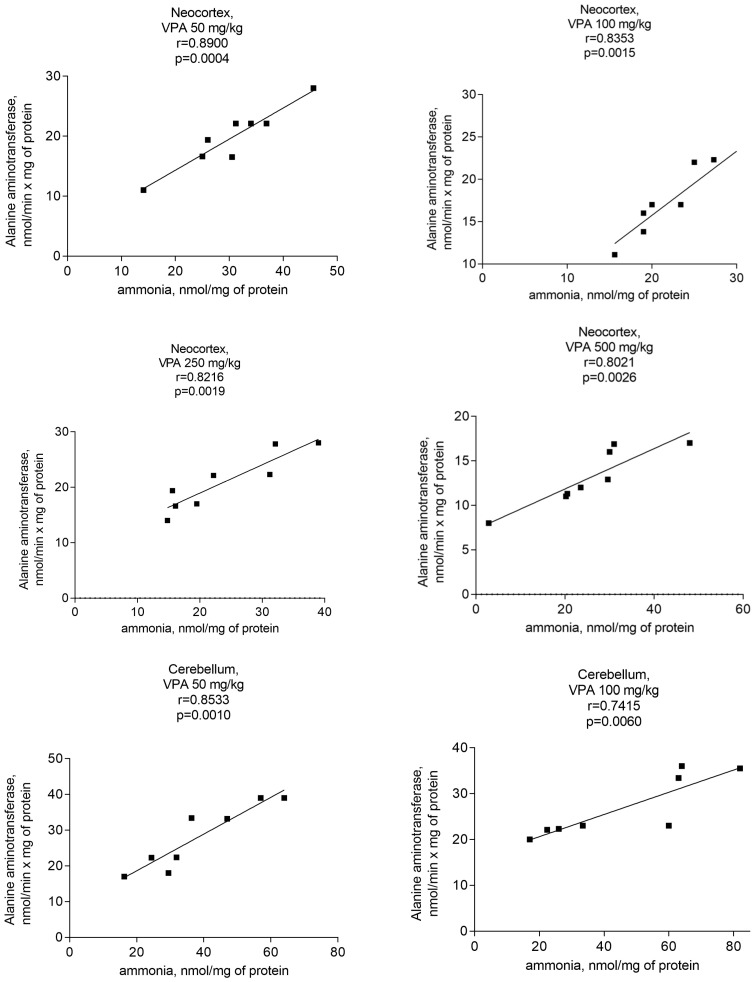

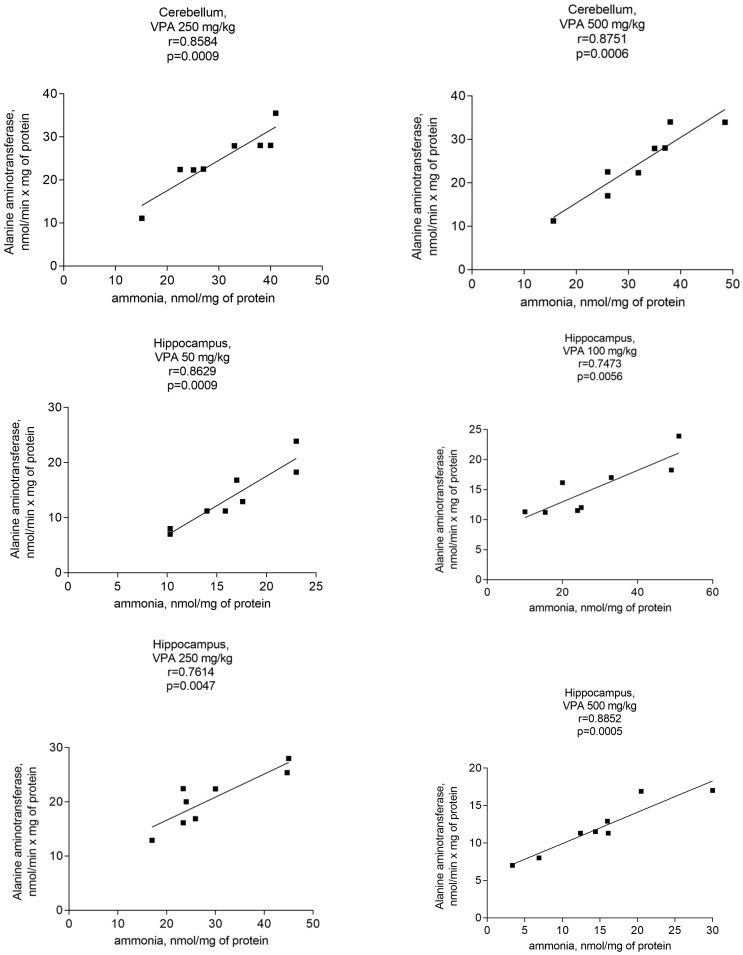
A positive correlation between mitochondrial ALT and ammonia in different brain areas of VPA-administered rats (50 mg/kg, 100 mg/kg, 250 mg/kg, and 500 mg/kg) (*r* = 0.7473–0.97770).

In addition, ALT activity was significantly and positively correlated with the mitochondrial level of ammonia (Figure 12).

The *r* values indicating that there is a correlation between the ALT activity and ammonia levels in all studied brain regions of the animals that received different doses of the drug varied from 0.740 to 0.89 (Figure 12) and were less pronounced than those for the GDH/ammonia (0.9040–0.9777 (Figure 9)), but were significant (*p* = 0.0004–0.0056).

These data highlight that the combined effects of GDH and ALT activities have a significant impact on the genesis of ammonia production in the mitochondria of the neocortex, cerebellum, and hippocampus, and that these enzymes, which are believed to be the main energy-sensor enzymes [58], are an important site for the functionally of significant VPA-associated brain energy damage in the drug-treated rats.

### 3.5. The Effect of VPA Treatment on Brain GS Activity

**Figure 13:**
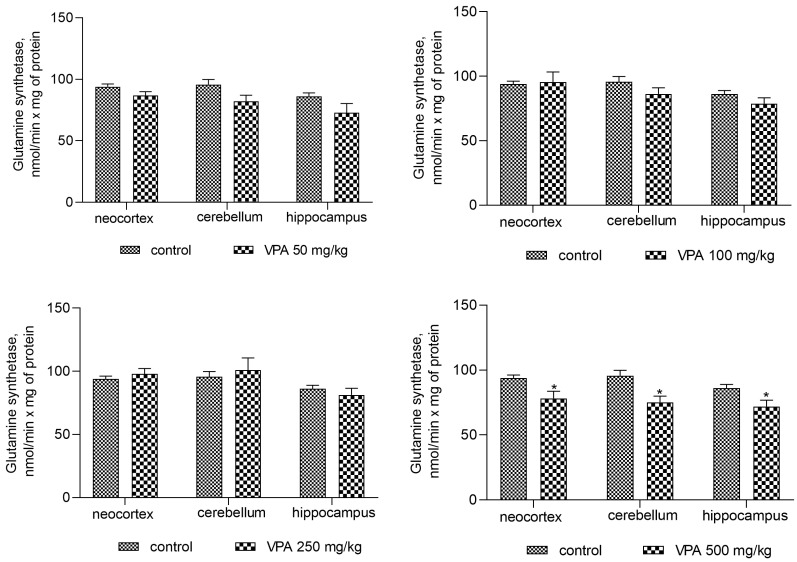
GS in the cytosol from different brain regions of the animals treated with 50 mg/kg, 100 mg/kg, 250 mg/kg, and 500 mg/kg VPA. The animals from the two groups were decapitated 30 min after VPA or saline injection. The results are the mean ± SEM of 8 rats per group. Values that are significantly different from those of the control group are marked with an asterisk: * *p* < 0.05 (with the Bonferroni correction for multiple comparisons).

Since ammonia arising in the brain through endogenous metabolic reactions is rapidly incorporated into glutamine by the action of glutamine synthetase and, therefore, ammonia accumulation in the brain can occur when the rate of its production exceeds the rate of its removal by GS [59], the purpose of our study was to investigate the effects of different doses of VPA on the activity of GS in rat brain regions (Figure 13).

The analysis showed that doses of 50 mg/kg, 100 mg/kg, and 250 mg/kg VPA did not lead to any noticeable change in GS activity in any of the studied areas of the animal brain (Figure 13). Further analysis of these three brain regions showed that an increase in the VPA doses of up to 500 mg/kg slightly but significantly decreased the enzyme activity by 16.7% (*p* = 0.0146), 21.4%, (*p* = 0.0380), and 16.5% (*p* = 0.0121) in the neocortex, cerebellum, and hippocampus, respectively, as compared to the control (Figure 13).

## 4. Discussion

Our data are the first direct evidence on fresh insights into the role that VPA plays in encephalopathy syndromes in the absence of liver disease after drug administration, even at therapeutic doses [60]. In the present study, it has been established that the activities of the enzymes involved in ammonia formation and neutralization are quite useful in studying the ammoniagenic effects of VPA on the rat brain. Thus, it was found that an increase in locomotor activity and anxiety, giving way to lower exploratory activity, slackness, drowsiness, tremors, and myoclonic seizures observed in the drug-treated animals developed when plasma ammonia and urea concentrations, as well as ALT and AST activities, were within normal limits (Table 1). To identify possible reasons for this phenomenon and whether VPA-induced neurological manifestations are accompanied by impaired ammonia homeostasis in the brain and thus contribute to its accumulation, our research goal has been to determine whether different doses of VPA affect the level of ammonia in the cytosol (Figure 1) and mitochondria (Figure 2) isolated from different areas of the rat brain. The analysis of the cytosol fractions of three brain regions under study showed that after VPA injection, ammonia levels increased dose-independently (roughly double, * p < 0.05) as compared to the control only in the cerebellum, and that none of the VPA doses had a significant effect on ammonia levels in the cytosol of the neocortex or hippocampus.

It is interesting that an increase in ammonia concentrations occurred together with a dose-independent significant elevation of only ADA activity in the cytosol of the cerebellum. This indicates that only in this brain area of the animals treated with different VPA doses, there was a significant correlation (*r* = 0.9–0.95, *p* < 0.0001–0.0003) between the ammonia levels and ADA activity.

The activity of other enzymes directly and indirectly involved in the formation of ammonia in the cytosol of brain cells, such as AMP-deaminase (*p* < 0.05–0.001, Figure 3) and AST (*p* < 0.05–0.001, Figure 4), was suppressed, and ALT was unaffected in all studied regions of the brain of rats treated with different doses of VPA. These results suggest that the effects of VPA on the ammonia-producing enzyme systems are region-specific, and that the VPA-induced elevation of ammonia levels in the brain could be related, at least in part, to the activation of ADA in the cytosol of the cerebellum. These findings are consistent with the results of our previously reported study showing that with increases in the concentrations of ammonia in the brain of rats with acute hyperammonemia, ADA activity increased only in the cerebellum [47]. Moreover, a strong correlation between the level of ammonia and the activity of this enzyme has been seen only in this region of the brain (out of the four regions of the brain studied) [61].

However, the brain ADA activity remains largely unexplored, the mechanism for the reported effect of VPA on the ADA upregulation, and the reasons for the differences between brain regions are enigmatic. Our previous research has found that the rat’s cerebellum appears to be most vulnerable to ammonium-induced oxidative stress in mild chronic HA-induced portacaval shunting [36], which is consistent with the differential responses of the cerebellum to hypoxia and reoxygenation [62], the age-related loss of cerebellar neurons [63], and the disturbance of Ca^2+^ homeostasis [64].

The cerebellum was recently reported to be involved in cognitive functions [65,66]. In addition, cerebellar damage resulting from its hypoperfusion and hypometabolism, an insufficiency of protective antioxidants, and oxidative stress processes [36] is known to be critically involved in disturbances of fine motor control and memory and language impairment [67].

Considering the importance of the relationship between an impaired brain energy metabolism and elevated ammonia production, and given that the cerebellum metabolic/energetic-dependent functions are affected during different pathologies, such as the VPA-associated inhibition of mitochondrial functions [16,17,68,69], the depression of the cerebral aerobic glucose metabolism [70], and an enhanced ATP degradation [71] resulting in a brain energy crisis, it might be implicated in the VPA-induced upregulation of ADA, leading to increased ammonia levels in this brain region. Just as importantly, an increase in ADA activity will inevitably lead to a decrease in the concentration of adenosine, which is one of the main neuroprotectors with pleiotropic action that control a myriad of functions and nerve cell vitality in general [72,73].

Steady states of brain ammonia concentration, however, also depend on the rate of its incorporation into glutamine by the action of GS, the only enzyme that directly removes ammonia in the brain [74]. In fact, there is our evidence that the activity of GS significantly decreases in the cytosolic fraction of all studied brain structures only under the influence of the highest dose of VPA (500 mg/kg, Figure 13), which is consistent with literature data that only high doses or the long-term use of VPA [75,76] create a situation where a decrease in the activity of this enzyme can be seen.

Glutamine synthetase is a highly oxidatively sensitive enzyme [77], and the decline in its activity accompanied by a faster increase in the rate of degradation [78] intensifies during the glucose deprivation associated with a large decrease in intracellular ATP levels [79].

In our previous study, it was shown that nitroarginine, a selective inhibitor of nitric oxide synthetase (NOS), significantly prevented the ammonia-induced disturbance to the brain energy metabolism and the death of rats with acute HA, and significantly increased GS activity [80,81], indicating that GS can be inhibited by NO formed by activated neuronal NOS in response to the ammonium-induced hyperactivation of NMDA receptors [82]. However, given that VPA usually exerts a dual-functionalized effect on the enzymatic activity [83,84], its effect on NOS activity has not been fully identified [85,86], and the conclusions are inconsistent [87], as with a more comprehensive understanding of the roles of NO in the dose-dependent VPA-induced GS downregulation in individual regions of the rat’s brain further study with more focus on the roles of NO is therefore suggested. At the mitochondrial level, in contrast to the cytosol, a high ammonia concentration was detected in all three brain areas of rats treated with VPA, but again the degree of accumulation of ammonia in the mitochondria (up to 100–200% above the control) did not depend on the dose of the drug used (Figure 2). To further elucidate whether the VPA-induced increase in ammonia levels in the mitochondria (Figure 2) changes in parallel with changes in enzymes involved in ammonia formation, the activities of glutaminase, GDH, ALT, and AST were determined.

The analysis showed that the concentration of ammonia in the mitochondria of the neocortex, cerebellum, and hippocampus of the animals exposed to various doses of VPA increased in parallel only to increases in the activity of GDH (Figure 8) and ALT (Figure 11) and strongly and positively correlated with the increased activity of these two enzymes (Figure 9 and Figure 12), while glutaminase activity did not change under these conditions, and AST activity significantly decreased as compared to the control in all studied brain regions (Figure 10). Apparently, an observed sharp and rapid decrease in the concentration of aspartate in the mitochondria of the brain in rats with acute HA [88] closely corresponding to the dynamics of the decrease in its concentration in the studied areas of the brain [57] in rats treated with VPA may be responsible for the suppression of AST observed under our experimental conditions.

The results, therefore, demonstrate that the increase in the content of ammonia in the brain mitochondria of animals treated with VPA occurred without the participation of glutaminase and AST, and that the main reason for increases of endogenous ammonia beyond a normal physiological threshold in these organelles in the absence of HA (Figure 2) is the combined effect of overactivated GDH and ALT.

GDH, as one of the known anaplerotic enzymes [58] that localizes to the mitochondrial matrix of nerve cells [89], which is controlled by the need of the cell for ATP, and depending on the availability of cofactors, ATP [90], and the ammonia concentration, either catalyzes the oxidative deamination of glutamate to α-ketoglutarate and ammonia, or participates in a reverse reaction of the binding of ammonia [9]. These findings are consistent with our data, suggesting that during the ammonia-induced inhibition of mitochondrial respiration and brain oxidative metabolism restriction [88,91], GDH displays an energy-sensing mechanism that serves as a source of energy that allows the enzyme to couple to the ALT in response to a decreased tricarboxylic acid cycle (TCA) function, thereby providing additional α-ketoglutarate into the cycle [92].

Considering the above, namely, that both the acute and chronic use of VPA can inhibit oxidative phosphorylation in cerebral mitochondria [15,35], it can be assumed that the VPA-induced activation of GDH and ALT is a compensatory mechanism aimed at restoring the energy metabolism, but this metabolic adaptation ensuring the availability of TCA cycle intermediates also leads to an increased production of ammonia, which, with reduced GS activity in the brain and a normally functioning liver, can be the culprit of encephalopathy. It should also be emphasized that since a significant part of the glutamate in astrocytic mitochondria undergoes oxidative degradation, limiting its availability for ammonium detoxification in the GS reaction [93], it can be assumed that the VPA-associated downregulation of GS in the conditions of impaired mitochondrial oxidative phosphorylation [94], among other things, may be associated with a lack of glutamate.

Taken together, these results indicate that in three regions of the rat brain, only some ammonia-producing enzymes were affected after VPA treatment, suggesting that the effects of VPA on ammonia-producing enzyme systems are region-specific and that a VPA-induced increase in the ammonia level in the brain beyond a normal physiological threshold and neurological alterations observed in the absence of liver failure may be associated with the activation of ADA in the cerebellar cytosol, while in the mitochondria, the main role in this process in all of the brain regions under study belongs to the combined action of GDH and ALT and at least, and partially to a decrease in GS activity, which occurs with the use of higher doses (500 mg/kg) of VPA.

Given that VPA-induced encephalopathy sometimes occurs in patients without signs of hepatic insufficiency, it is obvious that the elucidation of the mechanisms responsible for the VPA ammoniagenic action is an important biomedical task that is important to focus on first.

## 5. Conclusions

This study highlights the importance of the ammoniagenic action of VPA in the rat brain, which can develop in the absence of HA. Ammonia homeostasis in the brain is maintained to a large extend by the action of linked GDH and glutamate-ketoglutarate-dependent aminotransferase enzymes that simultaneously regulate both the steady state of exogenously-derived ammonia and energy production in the TCA cycle.

Based on the importance of the relationship between increased brain ammonia production and energy metabolism disorders [34], which together are predisposing factors in many neurodegenerative diseases [95], and based on the fact that different brain areas, which perform different physiological functions, may differ in their ability to detoxify and form ammonia, and, therefore, that the enzymes in each brain area have different sensitivities to VPA-related ammoniagenic effects, it is clear that prospective study is needed to explore how different brain areas alter in response to the administration of VPA in animals showing a compromised energy metabolism.

These studies will help to identify the risk factors for the development of one of the most dangerous VPA-induced adverse reactions associated with ammonium-induced encephalopathy that can develop even when the liver function is normal. Taking into account all pros and cons (disturbed energy metabolism → anaplerosis → GS-downregulation → increased ammonia levels beyond physiological thresholds), it is possible to create a framework for the development of innovative drugs with minimal adverse effects that will significantly expand the arsenal of medical supplies and improve the quality of medical services to minimize severe seizures in epilepsy patients.

## Figures and Tables

**Table 1:** Plasma concentrations of ammonia and urea as well as activities of ALT and AST in the blood plasma of animals after various single doses (50–500 mg/kg) of VPA (VPA group) or saline (control group) injection. The animals in both groups (*n* = 8 per group) were decapitated 30 min after VPA or saline injection. Plasma parameters were determined as indicated in the Materials and Methods section. Data represent the mean ± SEM. Significant differences in the values obtained from the “VPA group” were estimated by Student’s *t*-test (with the Bonferroni correction for multiple comparisons), ns—non-significant differences.

	Control	VPA, 50 mg/kg	VPA, 100 mg/kg	VPA, 250 mg/kg	VPA, 500 mg/kg
Ammonia, µM	200.5 ± 35.22	196.2 ± 29.64 ns	177.5 ± 17.59 ns	171.4 ± 21.57 ns	164.0 ± 13.74 ns
Urea, mM	6.91 ± 0.7	7.18 ± 11 ns	6.72 ± 0.59 ns	6.4 ± 0.72 ns	6.23 ± 0.96 ns
ALT, μmol/min × L	24.1 ± 9.8	25.83 ± 9.8 ns	26.34 ± 2.7 ns	24.36 ± 4.5 ns	26.17 ± 3.6 ns
AST, μmol/min × L	26.3 ± 3.8	27.95 ± 9.6 ns	30.03 ± 4.4 ns	26.8 ± 4.9 ns	25.7 ± 4.7 ns

## Data Availability

Data are contained within this article.
